# Seroprevalence of hepatitis B and C in Nepal: a systematic review (1973–2017)

**DOI:** 10.1186/s41124-018-0039-2

**Published:** 2018-09-06

**Authors:** Marcelo Contardo Moscoso Naveira, Komal Badal, Jagadish Dhakal, Neichu Angami Mayer, Bina Pokharel, Ruben Frank Del Prado

**Affiliations:** UNAIDS Nepal, UN House, Pulchowk, Lalitpur, GPO 107 Nepal

**Keywords:** Viral hepatitis, Hepatitis B, Hepatitis C, Systematic review, Epidemiology, Nepal

## Abstract

**Introduction:**

Hepatitis B and C represent an important co-infection for people living with HIV worldwide. Nepal wants to be part of the international mobilization for viral hepatitis elimination, and has pursued better understanding of the epidemic in its territory through scientific research.

**Methods:**

We performed a systematic review of seroprevalence studies hepatitis B and C in Nepal following the PRISMA 2009 Flow Diagram.

**Results:**

Fifty-four scientific publications and reports were selected for this review. Nearly a quarter of these documents have been issued in recent years and many are authored by non-governmental organizations in Nepal. The collective of information displays a wide range of alarming prevalence rates, particularly for girls and women survivors of human trafficking and a progressive participation of civil society in viral hepatitis epidemiology research in the country.

**Conclusion:**

This paper presents a most complete review of hepatitis B and C and HIV co-infection prevalence studies in different population groups from 1973 to 2016. A comprehensive analysis of the epidemiology and apparent trends in public health research and policy making in Nepal are also addressed in this document. We expect this to be a most important tool for improvements in future interventions for both epidemics in the country.

## Background

Viral hepatitis has become a leading cause of death and disability worldwide - estimated to be responsible for over 1.4 million deaths every year. Chronic viral hepatitis, mostly represented by the hepatitis B and C viruses infections (HBV and HCV, respectively), is a major cause of increasing events of high morbidity and mortality such as cirrhosis, end-stage liver disease and hepatocellular carcinoma. Both viruses are more easily transmissible than HIV [1].

The Sustainable Development Goals (SDG) establishes the year of 2030 as a desirable deadline for the end of many epidemics, including viral hepatitis. Nepal, a landlocked central Himalayan country in South Asia, has committed to the seventeen ambitious goals of SDG, and pursues to graduate from the least developed country rank by 2022. Nepal already presented remarkable achievements in infectious diseases, particularly the HIV response [2–4]. However, the understanding of viral hepatitis impacts to the country is limited. There is no national plan devised for the elimination of viral hepatitis and hepatitis C has only been briefly mentioned in the National HIV Strategic Plan 2016–2021 (Nepal HIVision 2020) [5].

Multiple community- and facility-based seroepidemiological surveys for viral hepatitis and co-infections have taken place in Nepal since 1973 [6–8]. Studies have assessed different population groups mostly in urban areas of Kathmandu Valley and to a lesser extent in other development regions.

As new National Guidelines for Viral Hepatitis are being developed, the Joint United Nations Programme on HIV/AIDS in Nepal (UNAIDS Nepal) understands this comprehensive review is a most welcome tool for future research. We expect this document to be useful for mathematical models, advocacy for key populations, improvements in public health policy, and setting priorities for successful elimination of hepatitis B and C.

## Methods

We conducted a systematic review of seroprevalence studies of hepatitis B and C following the PRISMA 2009 Flow Diagram [9]. Our main sources of data for this research were: 1) PubMed (Medline), through the following search expression “((“Hepatitis B”[Mesh] OR “Hepatitis B, Chronic”[Mesh] OR “Hepatitis B virus”[Mesh] OR “Hepatitis B Surface Antigens”[Mesh] OR “Hepatitis B Antibodies”[Mesh] OR “Hepatitis B”[Text] OR “Hepatitis C”[Mesh] OR “Hepatitis C, Chronic”[Mesh] OR “Hepatitis C Antibodies”[Mesh] OR “Hepatitis C”[Text]) AND (“Nepal”[Mesh] OR “Nepal”[Text]))”; 2) reports provided by the Government of Nepal (GoN); 3) reports authored by international agencies and non-governamental organizations (NGOs); and 4) personal correspondence to authors.

### Study selection

Two researchers took part in all steps of the reviewing process. We assessed our initial search results for eligibility through title, abstract and full-text analysis. Duplicates were not identified, but two publications were found to be supplemental to previously evaluated studies. One review obtained during the search presented additional data for three studies unavailable in digital media. Personal correspondences were sent to authors to obtain additional information. We could not identify any repetition of datasets.

Publications were considered eligible for inclusion if they presented own and original data (absolute numbers or percentage) for any population group, Nepalese or residing in Nepal, at any given site and time for at least one of the following outcomes of interest: 1) hepatitis B seroprevalence, active infection or exposure; and 2) hepatitis C seroprevalence as detected by anti-HCV tests.

Selected publications were excluded if full-text material could not be retrieved, if published before 1981 and if abstract could not provide sufficient information for any of the three outcomes of interest.

### Data extraction

The following data were then extracted from each eligible study included in this review: year of publication, population group, site, month and/or year of data collection, sample size, numbers/percentage of positive results for hepatitis B, C, HIV and syphilis; and authors’ name.

Seven studies did not provide details of which tests were used to define active HBV infection [7, 10–15]. One study identified did not provide details about which tests were used to define seroprevalence of exposition to HBV [16].

We chose to display results for every study total population and for as many subgroups as possible. Figures were obtained through full-text analysis and personal correspondence with authors. All numbers were thoroughly revised.

## Results

**Fig. 1:**
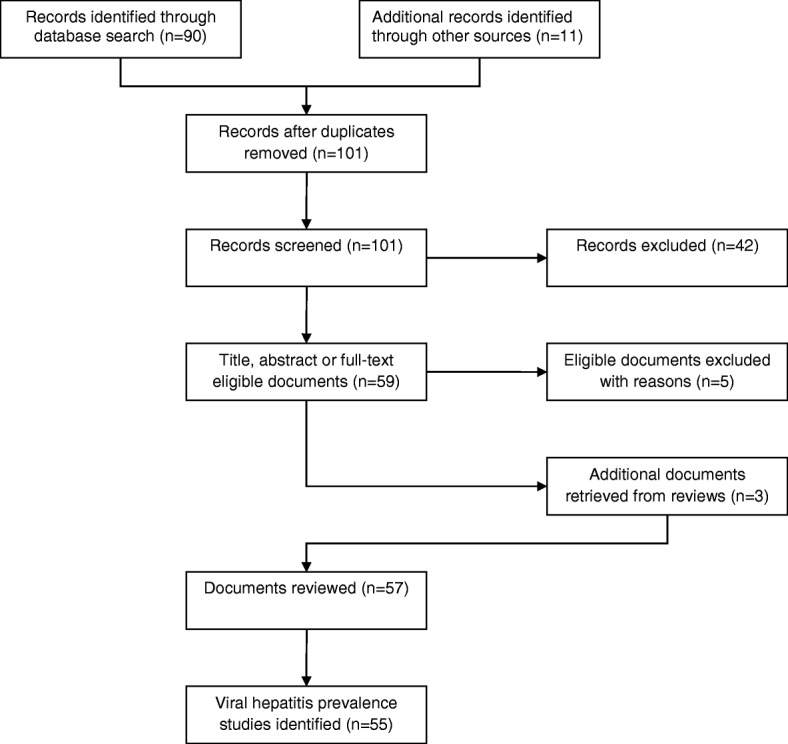
Flow of article selection for the viral hepatitis B and C prevalence studies in Nepal

This review selection process is depicted in Fig. 1, as adapted from the PRISMA 2009 Flow Diagram [9]. Initial search expression resulted in ninety different records with no duplicates. Forty-two citations were excluded after title, abstract and full-text screening and one citation was found to be supplemental. One report from Asian Network of People Who Use Drugs (ANPUD), one from HEPA Foundation/United Nations Office on Drugs and Crime (UNODC), one from United Nations Development Program (UNPD), two from Nepal Red Cross Society (NRCS) and six from Ministry of Health of Nepal (MoH), including the Global AIDS Response Progress Report 2015 (GARRP), were added to the group of forty-eight eligible citations, resulting in fifty-nine documents.

Five records were excluded because full-text could not be retrieved and their abstracts did not have data on any of the three outcomes of interest. Three additional documents from 1987 to 1990, which were not listed in PubMed, were identified from a review and later included in the collective. Fifty-seven documents relevant to fifty-five relevant prevalence studies were selected for this review.

Table 1 presents the collective of viral hepatitis prevalence studies with stratified population groups according to WHO key terms.

**Table 1:** Studies reporting hepatitis B and C in Nepal

SN	YEAR	POPULATION, SITE, TIME	SAMPLE SIZE, N	ANTI-HIV(%)	HBV			ANTI-HCV	NOTES	AUTHOR(S)
					**HBSAG(%)**	**ANTI-HBC(%)**	**ANTI-HBS(%)**			
1	1973	Patients attending hospitals in Kathmandu during infectious hepatitis outbreak, January 1973 to October 1973	53 sera samples	–	0 (0.00)	–	–	–	–	Hillis A et al. [6]
2	1984	Hospitalized patients with jaundice referred to Shree Tribhubn Chandra Military Hospital, Kathmandu and Infectious Disease Hospital, Teku, 1981 to 1982	41 patients	–	6 (7.50)	–	–	–	–	Kane MA et al. [28]
		Outpatients referred to Shree Tribhubn Chandra Military Hospital, Kathmandu and Infectious Disease Hospital, Teku, 1981 to 1982	39 patients	–		–	–	–	–	
3	1987	Children age 0–10 years, Surkhet Valley	45 children^a^	–	(6.6)	–	(22.2)	–	–	Shreshta SM. [72]
		Children, teenagers and adults age 11–20 years, Surkhet Valley	65 children, teenager and adults^a^	–	(3.0)	–	(46.6)	–	–	
		Adults age 21–40 years, Surkhet Valley	82 adults^a^	–	(9.7)	–	(44.0)	–	–	
		Adults 41+ years, Surkhet Valley	33 adults^a^	–	(6.0)	–	(25.0)	–	–	
		Girls and women, 0–41+ years, Surkhet Valley	–	–	(9.8)		–	–	–	
		Boys and men, 0–41+ years, Surkhet Valley	–	–	(4.4)	–	–	–	–	
		General population age 0–41+, Surkhet Valley	225 people^a^	–	(6.6)	–	(35.0)	–	–	
4	1987	Tibetan children age 0–9 years, Hemza, Tashi Ling, Pokhara; Jawalkhel, Kathmandu Valley	34 children	–	(20.0)	(32.0)^a^	–	–	–	Shrestha SM. [30]
		Tibetan children, teenagers and adults age 10–19 years, Hemza, Tashi Ling, Pokhara; Jawalkhel, Kathmandu Valley	79 children, teenagers and adults	–	(10.0)	(39.0)^a^	–	–	–	
		Tibetan adults age 20–29 years, Hemza, Tashi Ling, Pokhara; Jawalkhel, Kathmandu Valley	106 adults	–	(18.0)	(55.0)^a^	–	–	–	
		Tibetan adults age 30–39 years, Hemza, Tashi Ling, Pokhara; Jawalkhel, Kathmandu Valley	61 adults	–	(20.0)	(46.0)^a^	–	–	–	
		Tibetans age 40–49 years, Hemza, Tashi Ling, Pokhara; Jawalkhel, Kathmandu Valley	95 adults	–	(15.0)	(36.0)^a^	–	–	–	
		Tibetans age 0–49 years, Hemza, Tashi Ling, Pokhara; Jawalkhel, Kathmandu Valley	375 people	–	(16.0)	(45.0)^a^	–	–	–	
		Nepalese children age 0–9 years, Hemza, Tashi Ling, Pokhara; Jawalkhel, Kathmandu Valley	113 children	–	(0.0)	(3.5)^a^	–	–	–	
		Nepalese children, teenagers and adults age 10–19 years, Hemza, Tashi Ling, Pokhara; Jawalkhel, Kathmandu Valley	198 children, teenagers and adults	–	(0.5)	(5.0)^a^	–	–	–	
		Nepalese adults age 20–29 years, Hemza, Tashi Ling, Pokhara; Jawalkhel, Kathmandu Valley	110 adults	–	(1.8)	(5.4)^a^	–	–	–	
		Nepalese adults age 30–39 years, Hemza, Tashi Ling, Pokhara; Jawalkhel, Kathmandu Valley	49 adults	–	(0.0)	(32.6)^a^	–	–	–	
		Nepalese adults age 40–49 years, Hemza, Tashi Ling, Pokhara; Jawalkhel, Kathmandu Valley	25 adults	–	(0.0)	(16.0)^a^	–	–	–	
		Nepalese adults age 50+ years, Hemza, Tashi Ling, Pokhara; Jawalkhel, Kathmandu Valley	45 adults	–	(2.2)	(7.8)^a^	–	–	–	
		Nepalese age 11–41+ years, Hemza, Tashi Ling, Pokhara; Jawalkhel, Kathmandu Valley	540 people	–	(0.7)	(36.0)^a^			–	
5	1988	Patients during 1983 enterically transmitted non A–non B hepatitis outbreak, Kathmandu Valley	150 patients	–	34 (13.60)^a^			–	–	Nuti M [10]
		Controls during 1983 non A–non B hepatitis outbreak, Kathmandu Valley	100 people	–				–	–	
6	1988	Lactating women from six villages of Kathmandu Valley	26 women	–	0 (0.00)	4 (15.38)	8 (30.77)	–	–	Reynolds RD et al. [73]
		Breastfed infants age 2–6 months from six villages of Kathmandu Valley	26 infants	–	–			–	–	
7	1989	Patients age 20–40 years attending four Kathmandu hospitals, Kathmandu, 1985	460 sera samples	0 (0.00)	5 (1.09)	64 (13.91)	–	–	–	Mertens T et al. [74]
8	1990	Children age 0–5 years	57 healthy children	–	0 (0.00)	–	5 (8.77)	–	–	Shrestha SM [75]
		Children and teenagers age 6–15 years	359 healthy children ad teenagers	–	8 (2.23)	–	15 (4.18)	–	–	
		Teenagers and adults age 16–41 years	1788 healthy teenagers and adults	–	16 (0.89)	–	158 (8.84)	–	–	
		Adults, 41+ years	351 healthy adults	–	2 (0.57)	–	35 (9.97)	–	–	
		Girls and women, 0–41+ years	1529 healthy women	–	9 (0.59)	–	76 (7.40)	–	–	
		Boys and men, 0–41+ years	1026 healthy men	–	17 (1.65)	–	138 (9.02)	–	–	
		General population, 0–41+ years	2555 healthy individuals	–	26 (1.02)	23 (0.90)	214 (8.4)	–	–	
9	1991	Spouses of HBsAg chronic carriers	34 people	–	6 (17.65)	–	15 (44.12)	–	–	Shrestha SM et al. [29]
		Offspring of HBsAg chronic carriers	73 people	–	15 (20.55)	–	17 (23.29)	–	–	
		Siblings of HBsAg chronic carriers	29 people	–	9 (31.03)	–	11 (37.93)	–	–	
10	1993	Children age 0–4 years, Gurkha Contingent, Singapore	177 children	–	64 (19.28)	161 (17.05)	213 (22.56)	–	–	Goh et al. [32]
		Children age 5–14 years, Gurkha Contingent, Singapore	155 children	–				–	–	
		Children and adults age 15–24 years, Gurkha Contingent, Singapore	227 children and adults	–	–			–	–	
		Adults age 25–34 years, Gurkha Contingent, Singapore	289 people	–	–			–	–	
		Adults 35+ years, Gurkha Contingent, Singapore	96 people	–	–			–	–	
		Gurkha Community members, Gurkha Contingent, Singapore	944 people	–	26 (2.75)			–	–	
11	1994	Patients with chronic hepatitis attending Bir Hospital, Kathmandu	20 patients	–	12 (60.00)	–	–	5 (25.00)	–	Shreshta SM et al. [26]
		Patients with cirrhosis attending Bir Hospital, Kathmandu	63 patients	–	25 (39.68)	–	–	9 (14.28)	–	
		Patients with hepatocellular carcinoma attending Bir Hospital, Kathmandu	62 patients	–	20 (29.41)	–	–	6 (9.68)	–	
		Patients with chronic hepatitis, cirrhosis or hepatocellular carcinoma attending Bir Hospital, Kathmandu	145 patients	–	57 (39.31)	–	–	20 (13.79)	–	
		Pregnant women attending Bir Hospital, Kathmandu	83 women	–	0 (0.00)	–	–	3 (3.61)	–	
		Medical or paramedical personnel serving at Bir Hospital, Kathmandu	296 people	–	5 (1.69)	–	–	10 (3.38)	–	
12	1994	Female sex workers, Kathmandu Valley	341 women	3 (0.88)	37 (10.85)^a^			–	Syphilis: 73 (21.41)	Bhatta P et al. [76]
13	1994	Villagers, Dharan, Sunsari; Pancha Kanya Village Development Committee, Ilam; Dhankuta Hile, Dhankuta; Basantapur Village Development Committee, Tehrathm	303 serum samples	–	0 (0.00)	–	6 (1.98)	–	–	Rai SK et al. [77]
14	1995	Villagers, Bhadrakali and Kotyang villages, 1987	676 blood samples	–	2 (0.29)	52 (7.69)	–	1 (0.15)	–	Nakashima K et al. [78]
15	1996	People who inject drugs	72 people	–	4 (5.55)	59 (81.94)	–	58 (80.55)	–	Shrestha SM et al. [25]
16	1997	People who use drugs	72 people	–	2 (2.78)	44 (61.11)^b^		43 (59,72)	–	Shrestha SM et al. [16]
		People with chronic kidney disease	41 people	–	1 (2.44)	6 (14.63)^b^		1 (2.44)	–	
		People with chronic liver disease	145 people	–	57 (39.31)	74 (51.03)^b^		12 (8.27)	–	
		HBsAg carriers	49 people	–	49 (100.00)	49 (100.00)^b^		0 (0.00)	–	
		Healthy individuals undergoing routine check-ups	181 people	–	9 (4.97)	46 (25.41)^b^		0 (0.00)	–	
17	1998	Patients age ≤ 15 years attending Amp Pipal Hospital, Gorkha district, 1993	101 children and adults	–	–	0/9 (0.00)	1/9 (11.11)	0/9 (0.00)	–	De Bruyn et al. [44]
		Patients age 16–25 years attending Amp Pipal Hospital, Gorkha district, 1993		–	–	0/34 (0.00)	0/34 (0.00)	4/34 (11.76)	–	
		Patients age 26–35 years attending Amp Pipal Hospital, Gorkha district, 1993		–	–	1/20 (5.00)	0/21 (0.00)	0/21 (0.00)	–	
		Patients age 36–45 years attending Amp Pipal Hospital, Gorkha district, 1993		–	–	2/14 (14.28)	2/13 (15.38)	0/13 (0.00)	–	
		Patients age 46–55 years attending Amp Pipal Hospital, Gorkha district, 1993		–	–	1/8 (12.50)	1/8 (12.50)	0/8 (0.00)	–	
		Patients age ≥ 56 years attending Amp Pipal Hospital, Gorkha district, 1993		–	–	3/10 (30.00)	4/10 (40.00)	0/10 (0.00)	–	
18	1999	Male villagers age ≤ 24 years from Bhadrakali and Kotyang villages, August 1996 to September 1996	44 boys and men	–	0 (0.00)	1 (2.27)	–	1 (2.27)	–	Sawayama Y et al. [40]
		Female villagers age ≤ 24 years from Bhadrakali and Kotyang villages, August 1996 to September 1996	43 girls and women	–	1 (2.32)	1 (2.32)	–	0 (0.00)	–	
		Villagers age ≤ 24 years from Bhadrakali and Kotyang villages, August 1996 to September 1996	87 people	–	1 (1.15)	2 (2.30)	–	1 (1.15)	–	
		Male villagers 25–34 years from Bhadrakali and Kotyang villages, August 1996 to September 1996	40 men	–	0 (0.00)	2 (5.00)	–	2 (5.00)	–	
		Female villagers 25–34 years from Bhadrakali and Kotyang villages, August 1996 to September 1996	37 women	–	1 (2.70)	2 (5.40)	–	0 (0.00)	–	
		Villagers 25–34 years from Bhadrakali and Kotyang villages, August 1996 to September 1996	77 people	–	1 (1.30)	4 (5.19)	–	2 (2.60)	–	
		Male villagers 35–44 years from Bhadrakali and Kotyang villages, August 1996 to September 1996	49 men	–	0 (0.00)	1 (2.04)	–	1 (2.04)	–	
		Female villagers 35–44 years from Bhadrakali and Kotyang villages, August 1996 to September 1996	41 women	–	0 (0.00)	1 (2.44)	–	0 (0.00)	–	
		Villagers 35–44 years from Bhadrakali and Kotyang villages, August 1996 to September 1996	90 people	–	0 (0.00)	2 (2.22)	–	1 (1.11)	–	
		Male villagers 45–54 years from Bhadrakali and Kotyang villages, August 1996 to September 1996	29 men	–	0 (0.00)	1 (3.45)	–	2 (6.90)	–	
		Female villagers 45–54 years from Bhadrakali and Kotyang villages, August 1996 to September 1996	44 women	–	0 (0.00)	2 (4.54)	–	1 (2.27)	–	
		Villagers 45–54 years from Bhadrakali and Kotyang villages, August 1996 to September 1996	73 people	–	0 (0.00)	3 (4.11)	–	3 (4.11)	–	
		Male villagers 55–64 years from Bhadrakali and Kotyang villages, August 1996 to September 1996	29 men	–	1 (3.45)	5 (17.24)	–	0 (0.00)	–	
		Female villagers 55–64 years from Bhadrakali and Kotyang villages, August 1996 to September 1996	39 women	–	2 (5.13)	6 (15.38)	–	1 (2.56)	–	
		Villagers 55–64 years from Bhadrakali and Kotyang villages, August 1996 to September 1996	68 people	–	3 (4.41)	11 (16.18)	–	1 (1.47)	–	
		Male villagers ≥65 years from Bhadrakali and Kotyang villages, August 1996 to September 1996	38 men	–	0 (0.00)	7 (18.42)	–	0 (0.00)	–	
		Female villagers ≥65 years from Bhadrakali and Kotyang villages, August 1996 to September 1996	25 women	–	0 (0.00)	4 (16.00)	–	0 (0.00)	–	
		Villagers ≥65 years from Bhadrakali and Kotyang villages, August 1996 to September 1996	63 people	–	0 (0.00)	11 (17.46)	–	0 (0.00)	–	
		Female villagers age 15–90 years from Bhadrakali and Kotyang villages, August 1996 to September 1996	229 women	–	4 (1.75)	16 (6.97)	–	2 (0.87)	–	
		Male villagers age 15–90 years from Bhadrakali and Kotyang villages, August 1996 to September 1996	229 men	–	1 (0.44)	17 (7.42)	–	6 (2.62)	–	
		Villagers age 15–90 years from Bhadrakali and Kotyang villages, August 1996 to September 1996	458 people	–	5 (1.09)	33 (7.20)	–	8 (1.75)	–	
19	2000	Healthy Nepalese Men from Eastern development region, October 1996 to March 1997	100 men	–	2 (2.00)	–	–	–	–	Manandhar K et al. [79, 80]
		Healthy Nepalese Men from Central development region, October 1996 to March 1997	100 men	–	3 (3.00)	–	–	–	–	
		Healthy Nepalese Men from Western development region, October 1996 to March 1997	100 men	–	4 (4.00)	–	–	–	–	
		Healthy Nepalese Men from Mid Western development region, October 1996 to March 1997	97 men	–	4 (4.12)	–	–	–	–	
		Healthy Nepalese Men from Far Western development region, October 1996 to March 1997	81 men	–	5 (6.17)	–	–	–	–	
		Healthy Nepalese Men from five different development regions, October 1996 to March 1997	478 men	–	18 (3.76)	–	–	–	17 < 40 yrs. old -	
20	2002	Nepalese men age 16–50 years who required medical check-ups for employment abroad, July 1999 to September 1999	2585 men	–	24 (0.93)	–	–	–	–	Bidya S [81]
21	2003	Boys and men < 19 years, Baba Medical Center, Kathmandu, September 2003 to June 2004	52 boys and men	0 (0.00)	0 (0.00)	–	–	–	–	Joshi SK et al. [34]
		Men age 20–29 years, Baba Medical Center, Kathmandu, September 2003 to June 2004	375 men	5 (1.33)	9 (2.40)	–	–	–	Syphilis: 4/545	
		Men age 30–39 years, Baba Medical Center, Kathmandu, September 2003 to June 2004	170 men	5 (2.94)	7 (4.11)	–	–	–		
		Men age 40+ years, Baba Medical Center, Kathmandu, September 2003 to June 2004	30 men	–	1 (3.33)	–	–	–	–	
22	2003	People who inject drugs, Siddhi Polyclinic, Dillibazaar, Kathmandu	400 people	–	–	–	–	342 (85.5)	–	Shrestha IL [38]
		Adults without history of injection drug use, Siddhi Polyclinic, Dillibazaar, Kathmandu	400 people	–	–	–	–	3 (0.75)	–	
23	2003	Candidates for blood donation at Blood Bank Centre, NRCS, Teaching Hospital, Bhairahawa, February 2001 to April 2003	1548 samples	2 (0.13)	7 (0.45)	–	–	2 (0.13)	–	Chander A et al. [18]
24	2004	Sherpa people age 15–66 years, Lukla, Solukhumbu district, 2004	103 people	–	2 (1.94)	25 (24.27)	23 (22.33)	–	–	Chiba H et al. [82]
25	2005	Bhutanese refugees, Beldangi 2 Extension Camp, March 1998 to July 1998	467 people	–	4 (0.86)	–	–	–	–	Shah BK et al. [83]
26	2006	Healthcare workers of Bir Hospital, Kathmandu, December 2001 to February 2002	145 people	–	2 (1.38)	21 (14.48)	–	–	–	Shrestha SK et al. [68]
27	2006	Male villagers, migrant-returnes from India and non-migrants, from five village development committees, Doti District, April 2001	149 people	1 (0.67)	16 (10.74)	–	–	–	–	Poudel K et al. [31]
28	2007	Patients with liver cirrhosis or hepatocellular carcinoma attending Liver Foundation Nepal clinic, January 1998 to January 2004	121 patients	–	48 (39.67)	85 (70.25)	–	15 (12.40)	–	Shrestha SM et al. [27]
		Patients with liver cirrhosis attending Liver Foundation Nepal clinic, January 1998 to January 2004	70 patients	–	20 (28.57)	47 (67.14)	–	6 (8.57)	–	
		Patients with hepatocellular carcinoma attending Liver Foundation Nepal clinic, January 1998 to January 2004	51 patients	–	28 (54.90)	38 (74.51)	–	9 (17.65)	–	
29	2008	Symptomatic people living with HIV/AIDS at Manipal Teaching Hospital, Pokhara, March 2004 to September 2005	54 patients	PLWH	1 (1.85)^a^			1 (1.85)	–	Dhungel BA et al. [11]
30	2008	Candidates for blood donation, NRSC, CBTS, hospital units/mobile camps all over Nepal, 2001 to 2002	72,459 blood samples	–	627 (0.86)	–	–	384 (0.52)	–	Karki S et al. [19]
		Candidates for blood donation, NRSC, CBTS, hospital units/mobile camps all over Nepal, 2002 to 2003	73,758 blood samples	–	911 (1.23)	–	–	417 (0.56)	–	
		Candidates for blood donation, NRSC, CBTS, hospital units/mobile camps all over Nepal, 2003 to 2004	76,647 blood samples	–	663 (0.86)	–	–	321 (0.41)	–	
		Candidates for blood donation, NRSC, CBTS, hospital units/mobile camps all over Nepal, 2004 to 2005	82,677 blood samples	–	644 (0.77)	–	–	373 (0.45)	–	
		Candidates for blood donation, NRSC, CBTS, hospital units/mobile camps all over Nepal, 2005 to 2006	103,067 blood samples	–	887 (0.86)	–	–	366 (0.35)	–	
		Candidates for blood donation, NRSC, CBTS, hospital units/mobile camps all over Nepal, 2006 to 2007	115,720 blood samples	–	430 (0.37)	–	–	628 (0.54)	–	
		Candidates for blood donation, NRSC, CBTS, hospital units/mobile camps all over Nepal, 2001 to 2007	524,328 blood samples	–	4162 (0.82)	–	–	2489 (0.47)	–	
31	2008	Sex trafficked women and girls assisted by Maiti Nepal, Kathmandu	246 women and girls	74 (30.08)	66/210 (31.43)^a^			–	Syphilis: 48%	Silverman J et al. [7]
32	2009	Candidates for blood donation at the Central Blood Transfusion Service, Nepal Red Cross Society (NRCS), Kathmandu, December 2006 to September 2007	33,255 individual samples	65 (0.19)	–			–	–	Karki S et al. [12]
		People living with HIV/AIDS diagnosed during blood donation at the Central Blood Transfusion Service, NRCS, Kathmandu, December 2006 to September 2007	65 individual samples tested positive for anti-HIV	PLWH	–			7 (10.76)	–	
33	2009	Pregnant women admitted in the ward of NMCTH, Kathmandu, 2001 to 2007	5602	–	18 (0.32)^a^			–	–	Shreshta P et al. [84]
34	2009	People who inject drugs on Oral Substitution Therapy (OST), Kathmandu Valley, June 2009	118 people	–	–	–	–	95 (80.50)	–	HEPA Foundation [85]
		People who inject drugs, Kathmandu Valley, June 2009	82 people	–	–	–	–	47 (57.32)	–	
35	2009	Male candidates for blood donation, NRCS, Central Blood Transfusion Service (CBTS), Kathmandu, March 2008 to September 2008	18,434 blood samples of male candidates	25 (0.13)	92 (0.50)	–	–	128 (0.69)	Coinfection HIV/HCV: 8/128	Shrestha AC et al. [20]
		Female candidates for blood donation, NRCS, Central Blood Transfusion Service (CBTS), Kathmandu, March 2008 to September 2008	3282 blood samples of female candidates	2 (0.06)	10 (0.30)	–	–	11 (0.33)	–	
		Candidates for blood donation age ≤ 20 years, NRCS, Central Blood Transfusion Service (CBTS), Kathmandu, March 2008 to September 2008	3310 blood samples	2 (0.06)	7 (0.21)	–	–	7 (0.21)	–	
		Candidates for blood donation age 31–30 years, NRCS, Central Blood Transfusion Service (CBTS), Kathmandu, March 2008 to September 2008	9818 blood samples	12 (0.12)	45 (0.45)	–	–	75 (0.76)	–	
		Candidates for blood donation age 31–40 years, NRCS, Central Blood Transfusion Service (CBTS), Kathmandu, March 2008 to September 2008	5763 blood samples	10 (0.17)	29 (0.50)	–	–	42 (0.72)	–	
		Candidates for blood donation age 41–50 years, NRCS, Central Blood Transfusion Service (CBTS), Kathmandu, March 2008 to September 2008	2433 blood sample	3 (0.12)	19 (0.78)	–	–	13 (0.53)	–	
		Candidates for blood donation age 51–60 years, NRCS, Central Blood Transfusion Service (CBTS), Kathmandu, March 2008 to September 2008	392 blood samples	0 (0.00)	3 (0.51)	–	–	2 (0.51)	–	
		Candidates for blood donation, NRCS, Central Blood Transfusion Service (CBTS), Kathmandu, March 2008 to September 2008	21,716 blood samples	27 (0.12)	102 (0.46)	–	–	139 (0.64)	–	
36	2010	Candidates for blood donation in Banke blood transfusion service, July 2006 to June 2007	5211	–	63 (1.21)^a^			6 (0.11)	–	Tiwari BR et al. [13]
		Candidates for blood donation in Kaski blood transfusion service, July 2006 to June 2007	5995	–	21 (0.35)^a^			10 (0.17)	–	
		Candidates for blood donation in Morang blood transfusion service, July 2006 to June 2007	5351	–	47 (0.88)^a^			14 (0.26)	–	
		Candidates for blood donation in Banke, Kaski and Morang blood transfusion services, July 2006 to June 2007	16,557	–	131 (0.79)^a^			32 (0.19)	–	
37	2011	People who inject drugs, age 18–40 years, 2011	40 people	–	–	–	–	7 (17.50)	–	ANPUD. [86]
38	2012	Children age 10–12 years born from April 2000 to April 2002, before hepatitis B vaccine introduction, Kathmandu, April 2012	1200 children	–	3 (0.25)	–	–	–	–	Upreti SR et al. [35]
		Children age 5–6 years born from April 2006 to April 2007, after hepatitis B vaccine introduction, Kathmandu, April 2012	2187 children	–	3 (0.14)	–	–	–	–	
39	2012	Patients with ascites attending Nepal Medical College Hospital (NMCTH), Kathmandu, September 2011 to February 2012	43 patients	–	–	–	–	2 (4.65)	–	Adhikari P et al. [87]
40	2013	People with one or more risk behaviors attending National Public Health Laboratory (NPHL), Kathmandu, November 2011 to May 2012	678 people	105 (15.49)	–	–	–	–	–	Ojha CR et al. [88]
		People living with HIV/AIDS diagnosed among study population, November 2011 to May 2012	105 people	PLWH	–	–	14 (13.33)	–	–	
41	2014	People living with HIV/AIDS attending B P Koirala Institute of Health Sciences (BPKIHS), Dharan; Society for Positive Atmosphere and Related Support to HIV and IDS (SPARSHA), Kathmandu; Sukhra Raj Tropical and Infectious Disease Hospital, Teku, Kathmandu, April 2010 to March 2011	108 patients	PLWH	4 (3.70)^a^			3 (2.78)	HBV/HCV: 0 (0.00)	Barnawal SP et al. [15]
		People who inject drugs living with HIV/AIDS attending BPKIHS, Dharan; SPARSHA, Kathmandu; Sukhra Raj Tropical and Infectious Disease Hospital, Teku, Kathmandu, April 2010 to March 2011	205 patients	PLWH	24 (11.71)^a^			137 (66.83)	HBV/HCV: 10 (4.88)	
42	2014	Women living with HIV/AIDS, 18 years or older, February to March 2010	136 women	PLWH	–	–	–	9 (6.61)	–	Poudel KC et al. [39]
		Men living with HIV/AIDS, 18 years or older, February to March 2010	183 men	PLWH	–	–	–	129 (70,49)	–	
		People who do not inject drugs living with HIV/AIDS, 18 years or older, February to March 2010	189 people	PLWH	–	–	–	13 (6.88)	–	
		People who inject drugs living with HIV/AIDS, 18 years or older, February to March 2010	130 people	PLWH	–	–	–	125 (95.15)	–	
		People living with HIV/AIDS, 18 years or older, February to March 2010	319 people	PLWH	–	–	–	138 (43.26)	–	
43	2014	Central Blood Transfusion Centre, 2012–2013	67,644 blood samples	45 (0.07)	150 (0.22)	–	–	317 (0.47)	Syphilis: 394 (0.58)	NRCS [21]
		Regional Blood Transfusion Centre, 2012–2013	47,733 blood samples	40 (0.08)	188 (0.39)	–	–	126 (0.26)	Syphilis: 284 (0.59)	
		District/Emergency Blood Transfusion Centre, 2012–2013	61,624 blood samples	35 (0.06)	195 (0.32)	–	–	121 (0.20)	Syphilis: 118 (0.19)	
		Hospital Blood Transfusion Unit, 2012–2013	12,320 blood samples	3 (0.02)	32 (0.26)	–	–	21 (0.17)	Syphilis: 8 (0.06)	
		Total, 2012–2013	189,321 blood samples	123 (0.06)	565 (0.30)	–	–	585 (0.31)	Syphilis: 804 (0.42)	
44	2014	Children age 0–15 years with acute hepatitis attending the liver clinic of Bir Hospital and Norvic International Hospital of Kathmandu, Kathmandu, January 2006 to December 2010	312 children	–	15 (4.81)^a^			–	–	Sudhamshu KC et al. [14]
45	2015	Women who inject drugs attending Recovering Nepal services submitted to HIV testing, Nepalgunj	3 women	0 (0.00)	–	–	–	0 (0.00)	–	Kinkel HT et al. [8]
		Men who inject drugs attending Recovering Nepal services submitted to HIV testing, Nepalgunj	76 men	6 (7.89)	–	–	–	18 (23.68)	–	
		People who inject drugs attending Recovering Nepal services submitted to HIV testing, Nepalgunj	79 people	6 (7.59)	–	–	–	18 (22.78)	–	
		Women who inject drugs attending Recovering Nepal services, Dharan; Biratnagar	69 women	9 (13.04)	3 (4.35)	–	–	17 (24.64)	–	
		Men who inject drugs attending Recovering Nepal services, Dharan; Biratnagar	72 men	12/70 (17.14)	5 (6.94)	–	–	50 (69.44)	–	
		People who inject drugs attending Recovering Nepal services, Dharan; Biratnagar	141 people	21/139 (15.11)	8 (5.67)	–	–	67 (47.52)	–	
		Women who inject drugs attending Recovering Nepal services submitted to HIV testing, Kathmandu; Lalitpur; Chitwan	28 women	0 (0.00)	1 (3.57)	–	–	2 (7.14)	–	
		Men who inject drugs attending Recovering Nepal services submitted to HIV testing, Kathmandu; Lalitpur; Chitwan	153 men	22/108 (20.37)	2 (1.31)	–	–	113 (73.86)	–	
		People who inject drugs attending Recovering Nepal services submitted to HIV testing, Kathmandu; Lalitpur; Chitwan	181 people	22/136 (16.18)	3 (1.66)	–	–	115 (63.53)	–	
		Women who inject drugs attending Recovering Nepal services, Nepalgunj; Dharan; Biratnagar; Kathmandu; Lalitpur; Chitwan,	100 women	9 (9.00)	4 (4.00)	–	–	19 (19.00)	–	
		Men who inject drugs attending Recovering Nepal services submitted to HIV testing, Nepalgunj; Dharan; Biratnagar; Kathmandu; Lalitpur; Chitwan,	301 men	40/254 (15.75)	10 (3.32)	–	–	181 (60.13)	–	
		People who inject drugs attending Recovering Nepal services submitted to HIV testing, Nepalgunj; Dharan; Biratnagar; Kathmandu; Lalitpur; Chitwan,	354 people	49 (13.84)	–	–	–	–	–	
		People who inject drugs attending Recovering Nepal services, Nepalgunj; Dharan; Biratnagar; Kathmandu; Lalitpur; Chitwan,	401 people	92/397 (23.17)	14 (3.49)	146 (43.89)	–	200 (49.87)	–	
46	2015	Patients attending Manipal Teaching Hospital, Pokhara, 2008 to 2013	25,708 individual blood samples	218 (0.85)	–	–	–	–	–	Supram HS et al. [89]
		People living with HIV/AIDS at Manipal Teaching Hospital, Pokhara, 2008 to 2013	218 individual blood samples	PLWH	7 (3.21)	–	–	9 (4.13)	–	
47	2015	Boys and men 15+ years who inject drugs, Sunsari, Morang and Jhapa districts, July 2015	360 boys and men	30 (8.33)	3 (0.83)	–	–	171 (47.50)	Syphilis: 4 (1.11); Syphilis History: 8 (2.22)	MoH/NCASC [90]
48	2015	Boys and men 16+ years who inject drugs, Kathmandu, Lalitpur and Bhaktapur districts, Kathmandu Valley, June 2015 to July 2015	340 boys and men	22 (6.47)	0 (0.00)	–	–	75 (22.06)	Syphilis: 0 (0.00); Syphilis History: 0 (0.00)	MoH/NCASC [91]
49	2015	Boys and men 16+ years who inject drugs, Pokhara Valley, June 2015 to July 2015	345 boys and men	10 (2.90)	6 (1.74)	–	–	45 (13.04)	-STI: 4 (1.16)	MoH/NCASC [92]
50	2015	People living with HIV/AIDS in Eastern Development Region,	140 people	PLWH	5 (3.57)	–	–	67 (47.86)	–	UNDP/DFID/CMDN [2, 93]
		People living with HIV/AIDS in Central Development Region,	137 people	PLWH	7 (5.11)	–	–	30 (21.90)	–	
		People living with HIV/AIDS in West Development Region,	203 people	PLWH	13 (6.40)	–	–	20 (9.85)	–	
		People living with HIV/AIDS in Midwest Development Region,	51 people	PLWH	0 (0.00)	–	–	12 (23.53)	–	
		People living with HIV/AIDS in Far West Development Region,	146 people	PLWH	5 (3.42)	–	–	3 (2.05)	–	
		People living with HIV/AIDS in all Development Regions,	677 people	PLWH	30 (4.43)	–	–	132 (19.50)	–	
		People who inject drugs living with HIV/AIDS in all Development Regions,	562 people	PLWH	8 (1.42)	–	–	91 (16.19)	–	
		Sex workers living with HIV/AIDS in all Development Regions,	–	PLWH	(0.1)	–	–	0 (0.00)	–	
		Migrant workers living with HIV/AIDS in all Development Regions,	–	PLWH	(1.0)	–	–	(1.8)	–	
		Gay, Lesbian and Transgender people living with HIV/AIDS in all Development Regions,	–	PLWH	(0.4)	–	–	(0.4)	–	
		Non most at risk population living with HIV/AIDS in all Development Regions,	–	PLWH	(1.3)	–	–	(1.0)	–	
51	2016	People who inject drugs with last 30-day frequent injection drug use attending rehabilitation centers, Kathmandu; Bhaktapur; Lalitupur; Sindupalchowk	167 people	–	–	–	–	20/87 (22.99)	–	Loewinger G et al. [94]
52	2016	Girls and women 16+ years who inject drugs, Kathmandu, Lalitpur and Bhaktapur districts, Kathmandu Valley, April 2016 to July 2016	160 girls and women	14 (8.75)	3 (1.87)	–	–	34 (21.25)	12 (7.50)	MoH/NCASC [95]
53	2016	Boys and men 15+ years who inject drugs, Rupandehi, Kapilvastu, Dang, Banke, Kailali and Kanchanpur districts	300 boys and men	7 (2.33)	5 (1.67)	–	–	24 (8.00)	Syphilis: 1 (0.33); Syphilis History: 5 (1.67)	MoH/NCASC [96]
54	2016	Patients who came in contact with HIV or other chronic liver disease and jaundice attending Teku Hospital, Tribhuvan University Teaching Hospital, NRCS	2700 patients	–	–	–	–	100 (3.70)	–	Nepal A et al. [97]
55	2016	Central Blood Transfusion Centre, 2014–2015	69,303 blood samples	21 (0.03)	192 (0.28)	–	–	224 (0.32)	Syphilis: 360 (0.52)	NRCS [22]
		Regional Blood Transfusion Centre, 2014–2015	42,511 blood samples	13 (0.03)	151 (0.35)	–	–	56 (0.23)	Syphilis: 115 (0.27)	
		District/Emergency Blood Transfusion Centre, 2014–2015	77,016 blood samples	27 (0.03)	227 (0.29)	–	–	119 (0.15)	Syphilis: 260 (0.34)	
		Hospital Blood Transfusion unit, 2014–2015	28,324 blood samples	7 (0.02)	47 (0.16)	–	–	23 (0.08)	Syphilis: 25 (0.09)	
		Total, 2014–2015	217,154 blood samples	68 (0.03)	617 (0.28)	–	–	422 (0.19)	Syphilis: 760 (0.35)	

^a^: Study describes seroprevalence of active HBV infection. Test(s) used in the survey is(are) not specified

^b^: Study describes seroprevalence of exposure to HBV. Test(s) used in the survey is (are) not specified

Table 2 presents an analysis of the reviewed data and Cochran’s Q tests performed by Weill Cornell Medical College in Qatar. Estimated prevalence and heterogeneity has been presented for five population groups: PWID, populations at intermediate risk, populations at low risk (general population), populations with liver-related conditions and special clinical populations [17].

**Table 2:** Findings of the meta-analyses for hepatitis C virus (HCV) prevalence measures

Populations at risk	Studies	Samples	HCV prevalence estimates		Heterogeneity measures			
	Total N	Total N	Mean (%)	95% CI	Q (*p*-value)^a^	*τ* ^2b^	I^2^ (confidence limits-%)^c^	Prediction interval (%)^d^
People who inject drugs	15	3140	45.17	26.34–64.73	1714.1 (< 0.0001)	0.1487	99.2 (99.0–99.3)	0–100
Populations at intermediate risk	12	4998	12.76	5.44–22.47	668.83 (< 0.0001)	0.0486	98.4 (97.9–98.7)	0–59.58
Populations at low risk (general population)	28	972,123	0.68	0.54–0.86	683.44 (< 0.0001)	0.2027	96.0 (95.1–96.8)	0.26–1.75
Populations with liver-related conditions	6	411	11.51	7.73–15.87	7.40 (0.1926)	0.0018	32.4 (0–72.7)	3.48–22.89
Special clinical populations	3	133	1.67	0–5.81	2.79 (0.2473)	0.0022	28.4 (0–92.6)	0–75.38

^a^Q: the Cochran’s Q statistic is a measure assessing the existence of heterogeneity in HCV prevalence estimates

^b^*τ*^2^: the estimated between-study variance in the double arcsine transformed proportions of the true HCV prevalence estimates. The back-transformed *τ*^2^ was not calculated as the methodology to do so is not currently available

^c^I^2^: a measure assessing the magnitude of between-study variation that is due to differences in HCV prevalence estimates across studies rather than chance

^d^Prediction interval: estimates the 95% interval in which the true HCV prevalence in a new HCV study will lie

### Population groups

Candidates for blood donation account for just seven prevalence studies and yet represent approximately 90 % of the population evaluated for viral hepatitis in Nepal since 1973. This overwhelming presence of candidates for blood donation in seroprevalence studies does not contribute to the understanding of populations at increased risk of HBV, HCV and HIV co-infection in Nepal as they rarely present seroprevalence rates higher than 1 % [12, 13, 18–22].

It is understood that people at increased risk of HBV, HCV and HIV co-infections should be properly represented in our review. We have succeeded to identify studies for most groups of interest: general population, children, adults, pregnant women, people who inject drugs (PWID), patients attending healthcare services, sexual and household contacts of people chronically infected by HBV, sex workers (SW), healthcare workers (HCW), migrant workers, refugees/displaced persons and survivors of human trafficking.

We found only one survey of viral hepatitis in lesbian, gay, bisexual or transgender population (LGBT), including men who have sex with other men (MSM); and another in people with history of incarceration. All collected documents only referred to drug use as injection and did not acknowledge people who use drugs (PWUD) or different methods of drug administration (e.g. smoking heroin). [23, 24].

### Hepatitis B

A disease preventable by vaccination, hepatitis B has been identified in our review in forty-six studies. HBsAg (surface antigen) positive tests had highest values in PWID (1.3–81.9%), [8, 25] patients with jaundice, chronic liver disease, cirrhosis or hepatocellular carcinoma (7.5–60%); [16, 26–28] sexual and household contacts of people chronically infected by HBV (6.6–31), [29] girls and women survivors of sex trafficking (30%), [7] Tibetan population living in Kathmandu Valley (10–20%) [30] and Nepalese people outside Nepal (2.7–19.3) [31, 32]. On the other hand, the overall prevalence of hepatitis B in Nepal is estimated at 0.9%, [33].

Children, adults and general population cohorts also present interesting ranges for figures of hepatitis B seroprevalence. Older age groups present higher values for HBsAg [20, 34] and children born after vaccine implementation display reduced disease prevalence. [35].

Unfortunately, only one document presents viral hepatitis prevalence in LGBT population (MSM included), but it lacks important information on sample size and number of positive tests.

Exposure to hepatitis B virus, defined by anti-HBc (antibody against core antigen), has been assessed only in one key population - PWID, in two studies nearly twenty years apart. In 1996, more than 80 % of PWID had positive results for anti-HBc [25] and in 2015, when less than 45 % had positive results for the same marker [8].

### Hepatitis C

One of the most important causes of morbidity [36] and mortality, particularly for people living with HIV (PLWH), [37] hepatitis C seroprevalence has been featured in thirty-one studies, in a total of approximately one million people in Nepal. Prevalence rates range from zero to more than 80%, with highest figures found in PWID (85.5 in males; 24.6 in females), [38] PLHIV (43.3) and patients with hepatocellular carcinoma (17.6). [27, 38, 39].

Regardless of key population status, different prevalence rates have been observed in males and females [40]. Statistically significant differences according to gender can be verified in studies by Shrestha AC et al., between male and female candidates for blood donation in 2009 (0.69 vs. 0.33, respectively), [20] and Kinkel HT et a, between male and female PWID attending Recovering Nepal services in 2015 (19.00 vs. 60.13).

Age has been fundamental to the design of hepatitis C public health policies in many countries. It is known that age can relate to many factors in the epidemic: year of introduction of the virus, availability of tests and distribution of contaminated blood products, cumulative exposure (such as injection drug use, unprotected sexual activity across adult life), and status of harm reduction strategies [41–43]. In Nepal, age and hepatitis C have only been featured in two studies. De Bruyn et al. found anti-HCV to be positive in 11.8% of patients age 16–65 years attending Amp Pipal Hospital at Gorkha district in 1993; and Sawayama Y et al. found 2.27% in female villagers 45–54 years from Bhadrakali and Kotyang villages in 1996 [40, 44].

### HIV co-infection prevalence

The first HIV prevalence study in Nepal dates 1989, [44] 1 year after the first case of HIV was detected in the country [45]. So far, eighteen prevalence studies also assessed at some time the HIV infection in their population, almost half a million people and roughly half of the population evaluated for HBV or HCV infection since 1985, the year of debut of anti-HIV ELISA tests.

As stated previously for overall population, candidates for blood donation represent the majority of the population tested for HIV co-infection in Nepal in the fifty-five viral hepatitis prevalence studies of this review. Yet, regardless of age and/or gender subgroups, candidates for blood donation have failed to present HIV seropositivity rates above 0.2% [20, 12, 18], constituting themselves a population of low prevalence for HIV co-infection. This data must not considered a proxy for general population, as candidates for blood donation have shown to be a “poor control group for non-genetic studies of diseases related to environmentally, behaviourally, or socially patterned exposures”, [46] but a reason to pursue further detection of viral hepatitis in priority populations.

As we evaluate the remaining studied population, we find that the highest rates of HIV infection found in this review do not belong to PWID, but sex trafficked girls and women, at an approximate 30% rate of infection in 2008 (74/246) [7). This is closely followed by PWID attending Recovering Nepal services, if accounted the participation of those with previously defined HIV status, with rates as high as 23.17% in 2015. Such findings are consonant with latest numbers of Family Health International data for HIV prevalence in PWID in Kathmandu (21% in 2009) [3] and increase of HIV and sexually transmitted infections in survivors of sex traffic, particularly for Nepalese girls and women [47–49].

## Discussion

This review collects all available surveys performed in Nepal or with Nepalese population. It provides relevant information to policy makers, researchers and activists.

### Developments of improved strategic information

It has been 44 years since the first viral hepatitis prevalence study took place in Nepal. Since then, sixty different publications, of which fifty-five are available in this review, have dedicated themselves to the better understanding of these epidemics.

Almost a quarter of these scientific publications and reports have been issued in the last 3 years. While there is still much to investigate, it is undeniable that Nepal’s civil society organizations and academia have been successful in their struggle to improve the reactive approach to viral hepatitis and HIV, shaping public health policy and visibility of key populations.

Superior strategic information and overall engagement to the epidemics lead to additional victories: the inclusion of LGBT issues in government policies, the return of harm reductions strategy in 2007, increase in donor funding for the response to HIV, and stronger ties between emerging and existing networks of key populations and representatives, healthcare professionals, academia, UNAIDS and GoN [50–55]. Such echoing common voice for change lead to the preliminary discussion of National Viral Hepatitis Guidelines and negotiation of licensed generic drugs for hepatitis C treatment at a fraction of prices offered to other developing countries [41, 56–59]. This recent collective represents a cornerstone for viral hepatitis in Nepal.

### Gaps and key populations

It would seem to be that the shared modes of transmission of viral hepatitis and HIV, and the resulting similar epidemiological profiles, could translate into one equally successful public health intervention for both epidemics.

This is hardly the truth. Regardless of improvements in blood safety, availability of harm reduction services and assistance to sexually transmitted and reproductive tract infections, with significant drops in HIV prevalence since the last decade, many key populations sustain subpar decrease in hepatitis C numbers. Such is the case of PWID in Nepal: hepatitis C exceeds three times the HIV prevalence in several cohorts.

Since 2000, PWID, PLHIV, sex workers and LGBT have figured in no less than seventeen different viral hepatitis prevalence studies, almost a third of studies collected in this review, contributing without precedents to the national strategic information and deeper understanding of the response to public health interventions. Additional publications also developed initial data for other population groups such as refugees/misplaced persons and survivors of sex traffic.

Our research could not retrieve any studies regarding transgender population, male sex workers or incarcerated persons in Nepal.

### Hepatitis B vaccine coverage and elimination of vertical transmission (EVT)

Hepatitis B is a highly contagious, yet preventable disease [60, 61]. In recent years, many countries have chosen to scale up maternal and newborn care in order to secure a generation ‘free of hepatitis B’.

Our review provided two studies depicting hepatitis B in infancy in Nepal. The first study states hepatitis B is responsible for approximately 5 % of the cases of acute hepatitis in children 0–15 years [14]. The second study describes observed benefits of hepatitis B vaccine introduction to children in Nepal in 2002.

While no routine for hepatitis B immunoglobulin in prevention of vertical transmission has been implemented in Nepal, the rates of acute hepatitis related to HBV infection in children are quite different than the prevalence values of HBV in pregnant women (0.32%), an indicator that perhaps household exposure during infancy, and not mother-to-child transmission, is the reason for new but likely overlooked exposure during infancy (households).

Commercial vaccines for hepatitis B have been available worldwide since 1981 [62]. In Nepal, however, it has only been introduced in 2002. After fifteen years and despite recommendations issued by GoN, the country still struggles to provide appropriate vaccine coverage [35, 63].

Official government reports state that from 2002 until 2009, third-dose hepatitis B vaccine coverage for children 12–23 months stood at approximately 80%. [63, 64] World Health Organization (WHO) and United Nations Children’s Fund (UNICEF) estimate improved coverage in 20,012 and 2013, slightly above 95%. [65] Such higher vaccine coverage numbers are not homogenous in Nepal - not all municipalities have immunization plans or appropriate structure at their disposal [63, 64]. Moreover, the intervention requires timely birth-dose vaccination for a most successful response, [66] and faces many other obstacles [67].

Hepatitis B vaccine is available to healthcare workers (HCW) in many countries. In Nepal, HCW are featured in two studies as an alternative to controls, with HBsAg seropositivity rates of 1.38 and 1.69% [26, 68]. These figures are lower than the ones presented by key populations, but still higher than those of candidates for blood donation. Further investigation reveals that HCW and students in Nepal have largely mishandled biosafety procedures while at high risk of exposure to the infective agent. Studies in tertiary care centers have shown frequent needle-stick and sharps-related injuries, and incomplete or fully ignored vaccination and post prophylaxis procedures [69–71].

Nevertheless, UNAIDS understands that the strengthening of the immunization plan and maternal health must be accompanied of a nationwide awareness campaign for HCW and future health professionals.

### Community and the strengthening of health systems

Communities were the first responders to the HIV epidemic, nearly thirty years ago. They have continuously played an essential role in development of research, health services and shaping of public health policy worldwide, expanding their activities to sustainable and affordable vaccines and medicines for viral hepatitis.

Moreover, these collectives of advocates, researchers, clients or providers have the ability to work unisonous with marginalized populations, increasing the reach and quality of health systems and health services, often detecting overlooked issues such as stigma and discrimination. Whether leading research or promoting health services, civil society engagement improves awareness, prevention, diagnosis and retention in care. This has been the case of both viral hepatitis and HIV epidemic.

In Nepal, these initiatives are considered to be just as important as developments provided by academia, government and international agencies. It is clear that these endeavors provide a unique opportunity to fill critical gaps such as strategic information, low immunization coverage rates and elimination of mother-to-child and household transmission.

## Conclusion

The present review illustrates different turning points in viral hepatitis and HIV co-infection epidemiology in Nepal. Since 1973, when the first study on viral hepatitis in the country was published, there have been many changes in the understanding of these epidemics.

These include the indirect effects of successful public health policies aimed towards HIV, such as the decrease of viral hepatitis prevalence in PWID, and their limitations, revealing overlooked population groups and issues in viral hepatitis that require public health policies of their own.

The review also allows one to witness the progressive scientific development by Nepalese researchers and institutions, and civil society representatives’ participation. Such collaboration correlates with increased number of studies and sample sizes in recent years including the survey of key populations, and will be fundamental for the success of the National HIV Strategic Plan 2016–2021 and achieving the SDG by 2030.
